# Prognosis of Thyroid Nodules in Individuals Living in the Zhitomir Region of Ukraine

**DOI:** 10.1371/journal.pone.0050648

**Published:** 2012-11-28

**Authors:** Naomi Hayashida, Yui Sekitani, Jumpei Takahashi, Alexander A. Kozlovsky, Oleksandr K. Gutevych, Aleksey S. Saiko, Nina V. Nirova, Anjela A. Petrova, Ruslan M. Rafalskiy, Sergey A. Chorny, Valery V. Daniliuk, Masanobu Anami, Shunichi Yamashita, Noboru Takamura

**Affiliations:** 1 Department of Global Health, Medicine and Welfare, Nagasaki University, Nagasaki, Japan; 2 Department of Radiation Medical Sciences, Atomic Bomb Disease Institute, Nagasaki University Graduate School of Biomedical Sciences, Nagasaki University, Nagasaki, Japan; 3 Center for International Collaborative Research, Nagasaki University, Nagasaki, Japan; 4 Gomel State Medical University, Gomel, the Republic of Belarus; 5 Zhitomir Inter-Area Medical Diagnostic Center, Korosten, Zhitomir, Ukraine; 6 Nagasaki Clinical Diagnostic Pathology, Nagasaki, Japan; The University of Texas M. D. Anderson Cancer Center, United States of America

## Abstract

**Objective:**

After the accident at the Chernobyl Nuclear Power Plant (CNPP), the incidence of thyroid cancer increased among children. Recently, a strong relationship between solid thyroid nodules and the incidence of thyroid cancer was shown in atomic bomb survivors. To assess the prognosis of benign thyroid nodules in individuals living in the Zhitomir region of Ukraine, around the CNPP, we conducted a follow-up investigation of screening data from 1991 to 2000 in the Ukraine.

**Patients and Methods:**

Participants of this study were 160 inhabitants with thyroid nodules (nodule group) and 160 inhabitants without thyroid nodules (normal control group) intially identified by ultrasonography from 1991 to 2000. All participants were aged 0 to 10 years old and lived in the same area at the time of the accident. We performed follow-up screening of participants and assessed thyroid nodules by fine needle aspiration biopsy.

**Results:**

Among the nodule group participants, the number and size of nodules were significantly increased at the follow-up screening compared with the initial screening. No thyroid nodules were observed among the normal control group participants. The prevalence of thyroid abnormality, especially nodules that could be cancerous (malignant or suspicious by fine needle aspiration biopsy), was 7.5% in the nodule group and 0% in the normal control group (*P*<0.001).

**Conclusions:**

Our study indicated that a thyroid nodule in childhood is a prognostic factor associated with an increase in the number and size of nodules in individuals living in the Zhitomir region of Ukraine.

## Introduction

On April 26, 1986, the most serious nuclear accident in history involving radiation exposure occurred at the Chernobyl Nuclear Power Plant (CNPP). According to the report issued by the World Health Organization, more than 4,000 thyroid cancer cases were diagnosed during 1986–2002 among those who were children or adolescents, age 0 to 17 years, at the time of the Chernobyl accident in Belarus, Ukraine, and the four most contaminated regions of Russia [1]. After the accident, about 160,000 children underwent medical screening between 1991 and 1996 at five centers around CNPP within the framework of the Chernobyl Sasakawa Health and Medical Cooperation Project [2]. By 2000, the participants who underwent medical screening in this project numbered approximately 200,000. Evaluation included ultrasound examination of the thyroid gland and measurement of serum free thyroxine (fT4) and thyroid-stimulating hormone (TSH) concentrations and titers of antimicrosome and antithyroglobulin antibodies. There was a dramatic increase in childhood thyroid cancer in areas around CNPP, suggesting that these areas are high-risk zones for radiation-induced thyroid cancer [2], [3].

In this Sasakawa project, children with abnormal ultrasonographic thyroid findings underwent fine needle aspiration biopsy (FNAB) followed by cytological evaluation for confirmation of diagnosis. In this project, a strong correlation was shown between the incidence of thyroid nodules and thyroid cancers [2]. Furthermore, a strong relationship between solid thyroid nodules and the incidence of thyroid cancer was shown in survivors of the atomic bombs dropped on Hiroshima and Nagasaki [4]. This suggests that radiation exposure might be a risk factor for malignancies in patients with thyroid nodules.

Data on the role of radiation in the development of thyroid nodules come from cohorts of medically irradiated patients, those exposed to fallout from nuclear testing, or those exposed to emissions due to nuclear weapons production [5], [6]. Also, Imaizumi *et al.* reported that among atomic bomb survivors in Hiroshima and Nagasaki, a significant linear dose-response relationship was observed for the prevalence of all solid nodules: not only malignant tumors, but also benign nodules and cysts [7]. Although these results suggest that both internal and external radiation exposure appear to increase the risk of development of thyroid nodules, prognosis in patients with benign thyroid nodules exposed to radiation, i.e., the possibility of “malignant transformation,” has not been evaluated.

We performed follow-up evaluations on individuals living in the Zhitomir region of Ukraine, around the CNPP, who were previously assessed for thyroid nodules by ultrasound and FNAB to evaulate the prognosis of benign thyroid nodules.

## Materials and Methods

### Participants

The total number of children who underwent thyroid ultrasonography from 1991 to 1996 in the Zhitomir region was 29,161 children in the Chernobyl Sasakawa Health and Medical Cooperation Project; the number of participants ranged from 1,489 to 7,786 each year. The total number of children who underwent thyroid ultrasonography in this project from 1997 to 2001 in the region was 11,307. In this Sasakawa project, all children with abnormal ultrasonographic thyroid findings underwent FNAB followed by cytological evaluation for confirmation of diagnosis. In the Zhitomir region, a thyroid nodule was found in 66 children until 1996, and another 256 children were identified until from 1997 to 2000. Thus, we expanded the examination year of the first thyroid ultrasonography at the Sasakawa project to 2000.

All study participants were selected from among the individuals who were lived in the Zhitomir region at the time of the CNPP accident and screened in the Sasakawa project, and who were still living in the same region. Of this population, some participants were outpatients of hospitals or clinics in the Zhitomir region and some participants were called by telephone. We completed the participant selection when we reached the planned numbers of subjects in the nodule group and the normal control group. One hundred sixty inhabitants who underwent thyroid ultrasonography and were found to have benign thyroid solid nodules as part of the Chernobyl Sasakawa Health and Medical Cooperation Project from 1991 to 2000 (first screening) in the Zhitomir region of Ukraine were included in this study as nodule group participants. Patients with a history of thyroid cancer at the time of the accident, Hashimoto’s disease or functional thyroid disease at the time of screening, a history of unknown thyroid diseases, or a history of surgery or radiation therapy for thyroid disorders were excluded when we selected participants. As a normal control group, 160 sex- and age-matched persons from the same geographic region with normal thyroid findings by ultrasonography conducted during the Sasakawa Project were identified at random. Participants in each group were matched in the follow-up period since the first screening (median period [interquartile range; IQR] 172 months [134–197] in each group). Hence, the ages of the participants at the time of the first screening were equal (mean ± standard deviation [SD] 15.3±3.4 years in each group).

**Figure 1:**
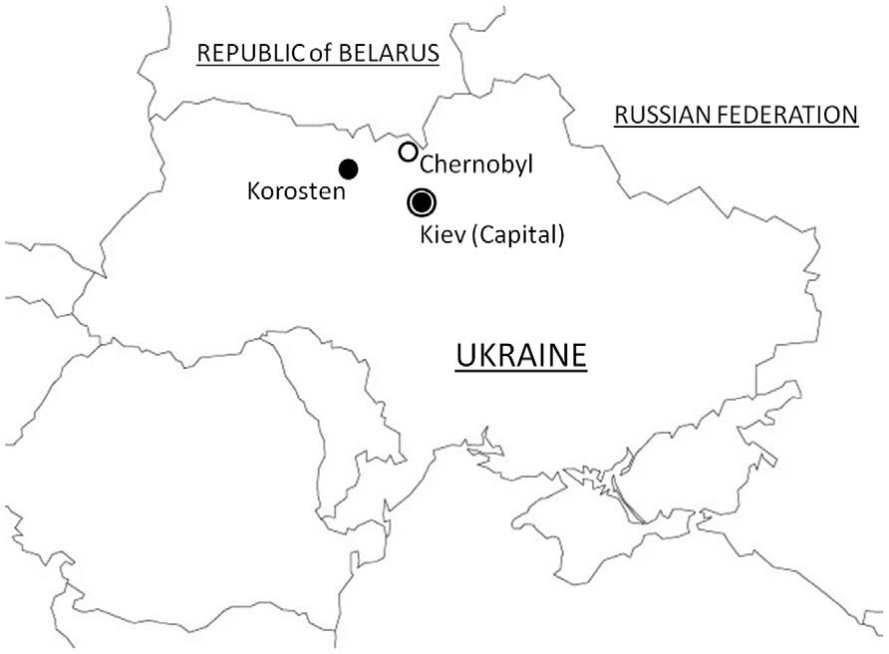
Location of Korosten, Ukraine relative to the Chernobyl Nuclear Power Plant (CNPP).

The study protocol was approved by the institutional review board of Korosten Inter-Area Medical Diagnostic Center, Korosten, Zhitomir, Ukraine. Informed consent was obtained from all participants. The location of Korosten city, Zhitomir region, relative to CNPP is shown in Figure 1. Contamination by radionuclides was remarkable in the regions to the northwest and west of the CNPP site. In Ukraine, the highest level of ^137^Cs contamination was observed in the region of Kiev and the Zhitomir and Rivne regions.

### First Screening

In the Sasakawa project from 1991 to 2000 (first screening), ultrasound examinations were performed with an Aloka SSD-520 or Aloka 630 ultrasonographic instrument (Aloka, Japan). Using an arch-automatic ultrasonographic instrument with a 7.5-MHz scanning probe, thyroid volume, position, structure, echogenity, and the presence of pathologic structures (such as nodules, cysts, and congenital abnormalities) were examined. In subject with abnormal ultrasonographic findings, FNAB was performed to confirm a diagnosis. Ultrasonographical abnormalities over 5 mm in diameter such as nodular lesions, cystic lesions, and abnormal echogenity were chosen as targets for FNAB. Fine needle aspiration biopsy was carried out with an echo-guided syringe pistol made to fit a 20-ml plastic syringe with a 22-gauge needle. In this study, we did not collect the laboratory data at the first screening.

### Ultrasonography

From May 2009 to December 2010, follow-up ultrasonography of the thyroid gland was performed both in the nodule and normal control groups (second screening). Ultrasonographic examination was conducted using 3.5– to 7.5-MHz probes (SSD-630 [Aloka, Japan] and Nemio XG SSA-580A [Toshiba, Japan]). In those cases in which nodules were identified, cross-sectional images were traced for measurement of size. The assessor recorded the size, location, and number of nodules. When nodules measuring more than 1 cm in diameter were detected by ultrasonography, cytological evaluation was performed from a sample obtained by FNAB. (Details of the FNAB procedure are described in the following section.) In cases in which study participants underwent operative treatment for their nodules, surgical and pathomorphological reports were reviewed.

### Fine Needle Aspiration Biopsy

Solid thyroid nodules underwent cytological or histological examination. When solid nodules measuring more than 1 cm in diameter were detected by ultrasonography, cytological examination was always conducted. Even if the size was under than 1 cm, we performed cytological or histological examination as possible. Among 320 participants, all participants with any thyroid nodules underwent FNAB under ultrasound guidance using a biopsy guide adaptor VAGL-007A (Toshiba, Japan). Fine needle aspiration biopsy was performed with a 22-gauge needle attached to a 20-ml disposable syringe with an aspirator. Written informed consent for FNAB was obtained from all participants before biopsy. Samples obtained were expelled on glass slides, smeared, and air-dried immediately for Giemsa staining. Every sample was examined by a cytologist in Korosten (Ukraine) and one in Nagasaki (Japan), from whom the details of the study participants were masked, to confirm the accuracy of their diagnosis. When the opinion was divided between the two observers, we decided to consult another Japanese cytological specialist about the cytological findings of the case. The results of FNAB were categorized into the following three groups: 1) benign, 2) suspicious, and 3) malignant. Benign results included normal thyroid tissue, adenoma, colloid nodules, and thyroiditis. Suspicious results included follicular and oxyphilic cell tumors. Malignant results included specimens with cytological atypia or cytological features of malignancy.

### Laboratory Analyses

Blood samples were collected from all 320 participants for measurement of fT4, TSH, thyroglobulin (Tg), antithyroglobulin antibodies (TgAb), and thyroid peroxidase antibodies (TPOAb). Serum fT4, TSH, Tg, TgAb, and TPOAb were measured using a Stat Fax® model 303+ enzyme-linked immunosorbent assay (ELISA) (Awareness Technology, Inc., Palm City, FL, USA) in the laboratory of the Korosten Inter-Area Medical Diagnostic Center. Inter-assay coefficients of variation were ≤ 10.8% for fT4, ≤ 10.8% for TSH, 5.4% to 7.1% for Tg, 3.9% to 8.5% for TgAb, and 5.7% to 7.1% for TPOAb. The laboratory reference ranges were 10.3–25.8 pmol/L for fT4, 0.3–6.2 IU/L for TSH, and ≤ 40 µg/L for Tg. Values <125 IU/mL for TgAb and <20 IU/ml for TPOAb were considered negative.

### Statistical Methods

**Figure 2:**
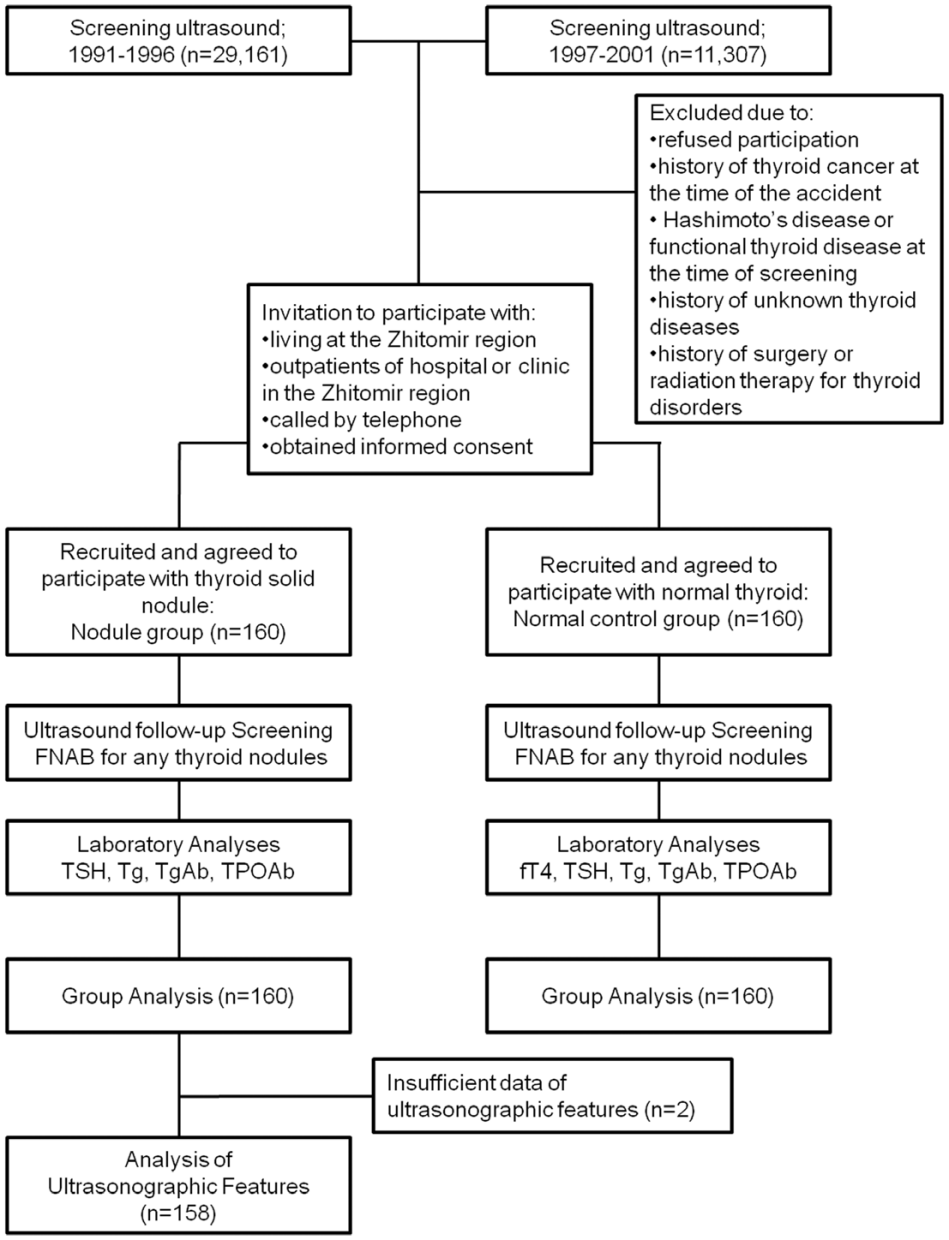
Flow chart of the study selection process and study method.

Data are expressed as mean ± SD or as median (IQR). In laboratory analyses, fT4 demonstrated a normal distribution, so the data was expressed as median ± SD. Because other laboratory data, such as TSH and Tg, the age of participants, the number of nodules, and the nodule diameter showed non-normal distribution, these data were expressed as median (IQR). Differences between the nodule and normal control groups were evaluated using Mann-Whitney’s U-test for age, gender, TSH, and Tg, and Student’s *t-*test for fT4. The chi-square test was used to investigate the difference between the groups in the prevalence of positivity for TgAb and TPOAb, and the frequencies of malignancies between groups. Wilcoxon’s test was used to compare numbers and sizes of nodules between the first (1991 to 2000) and second (2009 to 2010) screenings. The correlations between nodule number and laboratory data in the second screening were evaluated using Spearman’s correlation. All statistical analyses were performed using SPSS software, v.17.0 for Windows (SPSS Japan, Tokyo, Japan). Probability values less than 0.05 were considered indicative of statistical significance. The study design is summarized in Figure 2.

## Results

### Participants

Characteristics of the study participants are shown in Table 1. There were no significant differences of age (median [SD] 29 years [26–32] in each group) and male-female ratio between groups (15 males and 145 females in each group). In our study, only one patient (0.6%) in the nodule group demonstrated hyperthyroidism, defined as fT4 concentration above the normal range (>25.8 pmol/L) as well as TSH levels below the normal range (<0.3 IU/L), and one participant (0.6%) in the nodule group demonstrated hypothyroidism, defined as fT4 concentration below the normal range (<10.3 pmol/L) as well as TSH levels above the normal range (>6.2 IU/L). Furthermore, 10 patients (6.3%) in the nodule group and 7 participants (4.4%) in the normal control group demonstrated subclinical hyperthyroidism, defined as normal fT4 level (10.3 to 25.8 pmol/L) and TSH levels below the normal range (<0.3 IU/L), and 1 patient (0.6%) in the nodule group and 4 participants (2.5%) in the normal control group demonstrated subclinical hypothyroidism, defined as normal fT4 level (10.3 to 25.8 pmol/L) and TSH levels above the normal range (>6.2 IU/L) (data not shown). There were no significant differences in fT4 concentration (16.47±4.38 pmol/L in the nodule group vs. 17.25±4.12 pmol/L in the normal control group, *P* = 0.11) or TSH concentration (1.42 IU/L [0.65–1.93] in the nodule group vs. 1.41 IU/L [0.95–1.98] in the normal control group; *P* = 0.19). Also, there were no significant differences between the groups in TgAb-positivity (39/160 [25%] in the nodule group vs. 45/160 [28%] in the normal control group; *P* = 0.47) and TPOAb-positivity (34/160 [21%] in the nodule group vs. 29/160 [18%] in the normal control group; *P* = 0.48). However, Tg was significantly higher by Mann-Whitney’s U-test in the nodule group than among the normal control group participants (15.1 µg/L [6.9–30.4] vs. 8.2 µg/L [4.7–17.4]; *P*<0.001).

**Table 1:** Characteristics of study participants.

	Nodule group(n = 160)	Normal control group(n = 160)	*P*
Age (y)	29(26–32)	29(26–32)	0.80
Age at first screening (y)	15.3±3.4		
Gender			
Male (n)	15 (9%)	15 (9%)	1
Female (n)	145 (91%)	145 (91%)	
fT4 (pmol/L)	16.47±4.38	17.25±4.12	0.11
TSH (IU/L)	1.42 (0.65–1.93)	1.41 (0.95–1.98)	0.19
Tg (µg/L)	15.06 (6.93–30.35)	8.19 (4.71–17.41)	<0.001
TgAb positive	39 (25%)	45 (28%)	0.47
TPOAb positive	34 (21%)	29 (18%)	0.48

Data are n (%), mean±standard deviation (SD) or median (interquartile range; IQR).

### Ultrasonographic Features

Findings from ultrasound examinations were recorded for all 320 participants. No nodules were observed in the normal control group participants. Because the nodule sizes of two participants had not been recorded at the first screening, we analyzed the ultrasonographic features of 158 participants in the nodule group. At the first screening during 1991–2000, 181 nodules with maximum diameters between 0.4 and 3.7 cm were found among 158 nodule group participants. During the second screening during 2009–2010, we identified 251 nodules with maximum diameters between 0.5 and 4.3 cm among 158 nodule group participants. Nodule number and size were significantly increased, shown by Wilcoxon’s test, in the second screening compared with the first screening (*P*<0.001). In the first screening, 142 (90%) participants had solitary nodules and 16 (10%) participants had multiple nodules. In the second screening, 109 (69%) participants had solitary nodules and 49 (31%) participants had multiple nodules (*P*<0.001). There was no difference between screenings in nodule number among participants with multiple nodules (*P* = 0.38) (Table 2). At the first screening, 79 nodules were smaller than 1 cm and 81 nodules were more than 1 cm, and at the second screening, 13 nodules were smaller than 1 cm and 147 nodules were more than 1 cm. Of these 158 nodule group participants, 151 (95.6%) were found to have increased nodule size. Only 3 participants (1.9%) with initially diagnosed nodules demonstrated a lack of change in the size of the thyroid nodule, and in 4 participants (2.5%), the nodule size slightly decreased during follow-up. The second screening showed significant correlation between serum Tg levels and the number of thyroid nodules (*γ* = 0.22, *P* = 0.005) and size (*γ* = 0.30, *P*<0.001) by Spearman’s correlation (Table 3). There was no correlation between the thyroid nodule number or size and participant’s age (*γ* = 0.07, *P* = 0.41 for nodule number and *γ* = 0.13, *P* = 0.11 for nodule size).

**Table 2:** Thyroid ultrasonography findings at first and second screening (n = 158).

Ultrasonography findings	first screening(1991–2000)181 nodules	second screening(2009–2010)251 nodules	*P*
Number of nodules (median*)	1 (1–6)	1 (1–10)	<0.001
Solitary nodule case (n)	142 (90%)	109 (69%)	<0.001
Multiple nodules case (n)	16 (10%)	49 (31%)	
2	12	29	0.38
3	3	10	
4<	1	10	
Nodule diameter (cm)	0.95 (0.40–3.70)	1.55 (0.50–4.30)	<0.001

Data are n (%), *median (minimum-maximum) or median (IQR).

**Table 3:** Spearman correlation coefficients for nodule number and laboratory data in second screening per participant (n = 158).

	Nodule number		Nodule diameter	
	*Γ*	*P*	*γ*	*P*
fT4	−0.02	0.82	0.03	0.65
TSH	−0.20	0.01	−0.09	0.26
Tg	0.22	0.005	0.30	<0.001
TgAb	−0.01	0.87	0.04	0.65
TPOb	−0.06	0.43	0.09	0.27

### Cytological and Histological Findings

Fine needle aspiration biopsy results were obtained from all 160 participants in the nodule group. Samples from 148 (92.5%) participants were diagnosed as benign, 9 (5.6%) as suspicious, and 3 (1.9%) as malignant. All cytological results were consistent between Belarusian and Japanese cytologists. Hence, there was no need to consult another cytological specialist about the cytological findings. Among nodules classified as suspicious, four underwent surgery and one was ultimately diagnosed as malignant. Fine needle aspiration biopsy was not performed among the normal control group participants.

In the nodule group, 5 participants underwent thyroid surgery. Of these 5 participants, 4 had nodules that were diagnosed as suspicious and 1 as benign. Of the 4 suspicious nodules, histological diagnosis was 1 papillary carcinoma, 1 eosinophilic tumor, and 2 adenomas. The benign nodule was found to be normal thyroid tissue.

At the first screening, 158 participants underwent FNAB according to the same biopsy criteria and adenomatous goiters were diagnosed. Only two participants did not undergo previous FNAB due to the small nodule diameter (0.4 cm each). In these participants, adenomatous goiters were diagnosed by ultrasonographic features at the first screening and diagnosed as benign by FNAB in this study.

### Frequencies Of Malignancies

**Figure 3:**
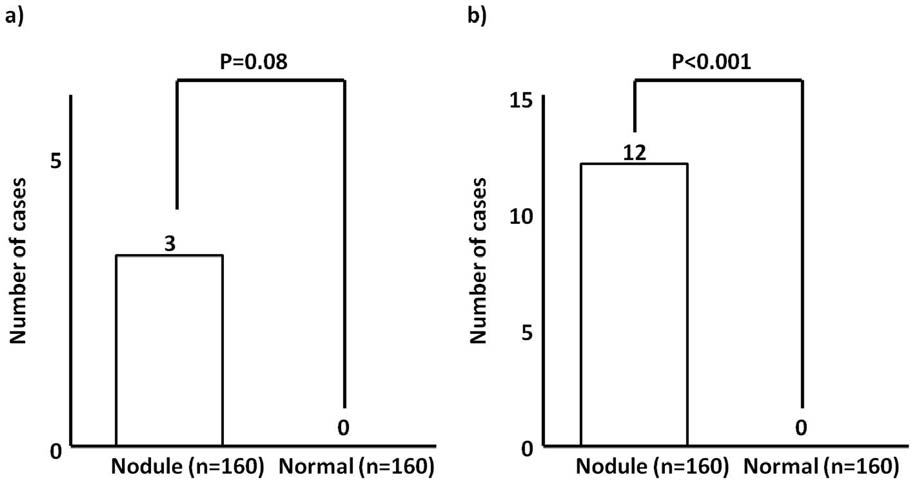
Number of cases confirmed as a) malignant and b) suspicious and malignant by fine needle aspiration biopsy.

Frequencies of malignancies are shown in Figure 3. Three nodule group participants had lesions diagnosed as malignant by FNAB. The frequency of malignancies (1.9%, 3/160) was not significantly higher when compared with the normal control group (0%, 0/160) (*P* = 0.08) (Figure 3a). Of these 3 participants with malignancy, 2 participants were female and 1 participant was male. There was no difference in the frequency of malignancies between male and female (*P* = 0.15). In addition, nine nodule group participants had lesions assessed as suspicious by FNAB. Of these 9 participants, 7 participants were female and two participants were male. As noted above, one female participant whose nodule was assessed as suspicious on FNAB underwent surgery and was confirmed to have a histopathologically malignant lesion. The frequency of suspicious or malignant FNAB diagnoses among nodule group participants (7.5%, 12/160) was significantly higher than among normal control group participants (0%, 0/160) by the chi-square test (*P*<0.001) (Figure 3b). There was no difference in the frequency of suspicious or malignant FNAB diagnoses between male and female (*P* = 0.05).

## Discussion

In this study, we evaluated the long-term risk of thyroid cancer development in irradiated individuals living in the Zhitomir region of Ukraine, who were 0 to 10 years of age at the time of accident. We found that the number and size of thyroid nodules in individuals who had thyroid nodules at the first screening were significantly increased at the second screening. By contrast, no thyroid tumor developed in individuals without thyroid nodules during the first screening. Furthermore, we performed FNAB on all patients with thyroid nodules and identified thyroid lesions among 12 participants that were potentially malignant, butthere was no significant difference between presence and absence of thyroid nodules. These results suggest that a thyroid nodule in childhood is not a significant risk factor for malignancy, but a prognostic factor associated with increase the number and size of nodules in individuals living around CNPP.

In this study, there were no differences in thyroid function and antithyroid antibodies between the nodule group and the normal control group. The Chernobyl Sasakawa Health and Medical Cooperation Project demonstrated that the prevalence of hyperthyroidism, defined as increased fT4 as well as decreased TSH levels, among 117,722 children was 0.14% and that of hypothyroidism, defined as decreased fT4 and increased TSH levels, was 0.13% [8]. In another study with a cohort of exposed children and adolescents from affected areas of the Ukraine, 0.6% of individuals were found to have hyperthyroidism [9]. Among atomic bomb survivors in Hiroshima and Nagasaki, hyperthyroidism was detected in 1.5% and hypothyroidism in 5.6% of 4,091 participants [7]. Previous studies reported the prevalence of hyperthyroidism and hypothyroidism was 0.1% to 2% for hyperthyroidism and 0.3% to 2% for hypothyroidism in the general population [10], [11], [12]. In our study, only one participant (0.6%) in the nodule group demonstrated hyperthyroidism and one participant (0.6%) in the nodule group demonstrated hypothyroidism. The rate of positivity for thyroid antibodies in our study was similar to the rates in previous reports with a disease-free, typical population [12], [13], but higher than a previous study in radiation-exposed children and adolescents reported 6 to 8 years after the CNPP accident [14].

Serum Tg levels were significantly higher in the nodule group than in the normal control group. It is well known that patients with thyroid nodules have higher serum Tg concentrations. Moreover, serum Tg concentrations are correlated with number and size of nodules [12]. Our study also showed a significant correlation between serum Tg levels and the number of thyroid nodules and their size. In this study, there was no correlation between the number of thyroid nodules or their size and the participant’s age. This finding suggests that age at the time of the Chernobyl accident may not influence the number and size of nodules.

Ultrasound examination showed nodule number and size in nodule group participants were significantly increased in our second screening compared with the first screening. Among evaluable 158 nodule group participants, 151 (95.6%) were found to have increased nodule size. There are few reports about the natural history of benign thyroid nodules in individuals living around Chernobyl nuclear power plant. Erdogan *et al*. reported about the natural course of benign thyroid nodules in a moderately iodine-deficient area [15]. In their report, a significant decrease in the largest dimension, defined as more than 50% during follow-up, was found in eight nodules (1.5%). In addition, a significant increase, defined as more than 50% during follow-up, was found in 22 nodules (4.2%). In our study in Korosten, also an iodine-deficient area, no nodule demonstrated more than 50% decrease and 106 participants (67%) had thyroid nodules that increased more than 50% during follow-up.

Erdogan *et al*. also reported that repeated biopsies in significantly increased nodules (i.e. >30% increase in volume) were all found to be benign [15]. In our study, 3 nodule group participants had lesions diagnosed as malignant and 9 had lesions diagnosed as indeterminate according to FNAB. As a result, the thyroid abnormality rate among the nodule group participants, that is, those with indeterminate and malignant lesions based on FNAB, was significantly higher than among the normal control group participants. In particular, these three nodules diagnosed as malignant were increased more than 50%. Furthermore, 7 of 9 indeterminate nodules were increased more than 30% and 5 indeterminate nodules had increased more than 50%. These results suggest that thyroid nodules in individuals living around CNPP are more likely to increase and a significant increase in the maximum diameter of thyroid nodules is a risk factor for the development of malignant tumors.

There have been many reports about thyroid cancer after the Chernobyl accident. A report about childhood thyroid cancer in Belarus, Russia, and Ukraine after Chernobyl showed that >10-fold maximal elevation in the incidence of thyroid cancer was observed roughly a decade later [16]. In a study of 276 patients with thyroid cancer who were younger than 15 years of age at the time of the Chernobyl accident, a strong dose-response relationship was observed between radiation dose to the thyroid received in childhood and thyroid cancer risk [17]. However, it has been reported that primary thyroid cancer developing after internal exposure to radioiodine after the Chernobyl accident does not display specific risk factors for recurrence different from those for sporadic primary thyroid cancer [18]. With regard to non-malignant thyroid disease, a significant linear dose-response relationship was observed for the prevalence of benign thyroid nodules among atomic bomb survivors in Hiroshima and Nagasaki [7]. Among children exposed to radiation after the Chernobyl accident, there was a significant correlation between the prevalence of benign thyroid nodules and malignant thyroid neoplasms [2]. To our knowledge, ours is the first report about the natural course of benign thyroid nodules after internal exposure to radioiodine from the accident at the CNPP. Our data suggest that a thyroid nodule in childhood after exposure to radiation from the CNPP is a risk factor for the future development of growth of nodules and thyroid cancer.

Our study has several limitations. First, we could not evaluate the histopathological findings of most participants. In this study, we evaluated the results of FNAB as benign, suspicious, or malignant. Findings of suspicious and malignant were regarded as malignancy. According to the current six-category diagnostic scheme (benign lesions, lesions of undetermined significance, follicular neoplasms, suspicious for malignancy, malignant, and unsatisfactory), lesions of undetermined significance, follicular neoplasms, and those suspicious for malignancy are classified as suspicious. In a review of thyroid fine needle aspiration, the risk of malignancy in this classification scheme was reported to be 5–10% in lesions of undetermined significance, 20–30% in follicular neoplasms, and 50–75% in those suspicious for malignancy [19]. Seningen *et al*. reported that, for thyroid malignancy as the disease outcome using cytologic thresholds of atypical, suspicious, and positive, overall sensitivity of FNAB was 94.5% for atypical findings, 94.1% for suspicious findings, and 65.0% for positive findings, and specificity was 46.0% for atypical findings, 48.3% for suspicious findings, and 98.5% for positive findings [20]. They also reported that the positive predictive value for all malignancies was 97.0% and the negative predictive value was 92.0%. However, accurate calculations of sensitivity, specificity, false-positive rate, and false-negative rates are difficult because the great majority of patients with nodules with benign cytology do not undergo surgery. Furthermore, the average percentage of nodules with inconclusive or indeterminate cytology obtained by FNAB guided by ultrasound is approximately 5% to 17% [21]. Based on final histopathology, 19.2% to 35.3% of indeterminate FNABs were diagnosed as malignant [22], [23], [24]. Thus, although benign lesions may be diagnosed as malignant or malignant lesions may be diagnosed as benign, these findings raise the possibility of malignancy among lesions with indeterminate FNAB results.

In this study, there is no significant difference between the nodule group and the normal control group with respect to the frequency of malignancy (3/160 [1.9%] vs. 0/160 [0%], *P* = 0.08). Furthermore, there is no significant difference in the frequency of malignancies between male and female. These may be due to the small number of participants. In the Chernobyl Sasakawa Project, the prevalence of thyroid nodules was higher in girls than in boys and the ratio of the prevalence in girls to that in boys ranged from 1 to 2 (mean 1.64). Furthermore, thyroid nodules are age-dependent, a fact that explains their higher incidence in older age groups [2]. In this study, we could not investigate the influence of other individual variables such as iodine intake or age. Another limitation is that we could not evaluate individual internal radiation doses. There are some reports concerning the relationship between the prevalence of thyroid nodules or carcinoma and radiation dose. A strong dose-response relationship was observed between radiation dose to the thyroid received in childhood and thyroid cancer risk after the Chernobyl accident [17]. Also, in Hiroshima and Nagasaki atomic bomb survivors, a significant linear dose-response relationship was observed for the prevalence of all thyroid nodules [7]. In a report concerning the prevalence of ultrasound-detected thyroid nodules and radiation dose, prevalence was significantly and independently associated with both external and internal doses [25]. Recently, Brenner *et al*. reported the results of ^131^I dose-response for incident thyroid cancers related to the Chernobyl accident [26]. They indicated that the dose-response relationship was linear and the excess relative risk (ERR) per gray was 1.91. In our study, although we included all participants from same region, we could not evaluate individual living conditions, especially usual eating habits, which might affect radiation dose. Because the radiation effect appears late, even if inhabitants around CNPP had any mutation with radiation, the follow-up period of this study may not be long enough to assess the effect of radiation. Also, we could not evaluate family history, age or intrinsic microcalicification of thyroid nodules in each individual which may affect on subsequent development of malignancy from thyroid nodules. Further investigations that include the assessment of internal radiation dose are needed to delineate the relationship between radiation dose and the prevalence of thyroid malignant tumors.

In conclusion, we used screening to assess the prognosis of benign thyroid nodules in individuals living around CNPP and found that a thyroid nodule in children lived in the Zhitomir region of Ukraine at the time of the CNPP accident is a prognostic factor associated with increase the number and size of nodules. On the other hand, it cannot be denied that the presence of thyroid nodules is a potential risk factor for the development of malignant tumors. Further studies are needed to confirm these results and to assess the influence of radiation dose on the long-term risk of cancer.
